# Non-invasive Quantitative Analysis of Specific Fat Accumulation in Subcutaneous Adipose Tissues using Raman Spectroscopy

**DOI:** 10.1038/srep37068

**Published:** 2016-11-15

**Authors:** Phiranuphon Meksiarun, Bibin B. Andriana, Hiroko Matsuyoshi, Hidetoshi Sato

**Affiliations:** 1 Department of Biomedical Chemistry, School of Science and Technology, Kwansei Gakuin University 2-1, Gakuen, Sanda, Hyogo, 669-1337 Japan

## Abstract

Subcutaneous adipose tissue (SAT), visceral adipose tissue (VAT), and fat beneath the dermis layer were investigated using a ball lens top hollow optical fiber Raman probe (BHRP). Hamsters were fed with trilinolein (TL) and tricaprin (TC) for six weeks and measurements were carried out every two weeks. The BHRP with an 800 μm diameter fused-silica ball lens was able to obtain information on the subcutaneous fat in a totally non-invasive manner. Changes in the concentration of TL and TC during the treatment were analyzed, and the relationship between fat accumulation and dietary fat was studied. It was found that SAT had, in general, a higher degree of unsaturation than VAT. The accumulation rate of TC found in SAT and VAT was 0.52 ± 0.38 and 0.58 ± 0.4%, respectively, while the TL accumulation rate was 4.45 ± 1.6 and 4.37 ± 2.4%, respectively. The results suggest different metabolic pathways for TC, a typical medium-chain fatty acid, and TL, a long-chain unsaturated fatty acid. Raman subsurface spectra were successfully obtained and used to analyze the subcutaneous fat layer. The accumulation rates of TL and TC found in skin fat were 5.01 ± 3.53% and 0.45 ± 0.36%, respectively. The results demonstrate the high feasibility of Raman spectroscopy for non-invasive analysis of adipose tissue.

Obesity is becoming a worldwide issue as societies develop. It is characterized by the significant accumulation of fat in the subcutaneous and visceral adipose tissue. It has been reported to induce various types of medical conditions, including diabetes and heart-related diseases. Visceral adipose tissue (VAT) abnormality has been reported to be directly correlated with numerous medical conditions, such as insulin resistance, diabetes, gallstone disease, and coronary heart diseases. Excessive visceral fat have been reported to be closely related to obesity and type II diabetes mellitus^1^. An important role of visceral fat – in addition to energy storage, glucose and lipid metabolism, and producer of a number of hormones – is strongly related to inflammation, which results in the high risk of cardiovascular disease associated with obesity^2^. The fat in subcutaneous adipose tissue (SAT) has also been reported to be involved in insulin resistance and inflammation in overfed humans^3^.

The lipids found in adipose tissue are mostly in the form of triacylglycerol (TAG). The type and amount of fatty acids in TAG can affect the lipid metabolic system in various ways, depending on their chain length and degree of unsaturation. The length of the fatty acid chains plays an important role in their metabolism: while medium-chain fatty acids are metabolized mostly in the liver^4^, the metabolic pathway for long-chain fatty acids is different, as they are mostly packed into chylomicrons and sent through the lymphatic system to peripheral tissues. The unsaturation degree of the fatty acids is another important factor. Unsaturated fatty acids have been believed to be beneficial for health for a long time. The C=C double bonds in the fatty acids play pivotal roles as signal messengers and lipid mediators in various biological systems. Unsaturated fatty acids have also been reported as anti-oxidation supplements. In contrast, saturated fatty acids have a negative impact on the saturated fat intake and have been reported to induce coronary heart diseases and obesity. Despite their harmful effects, medium-chain saturated fatty acid species can help patients diagnosed with malabsorption syndrome.

The development of analytical techniques for adipose tissues has contributed to monitoring the condition and properties of lipids for biomedical applications. A series of methods have been proposed for measuring the body mass index or the waist circumference using dual-energy X-ray absorptiometry and computed tomography^5^,^6^. These approaches have been used to determine the size, structure and location of SAT and VAT. Raman spectroscopy has been used in several studies for the determination of adipose tissue, as this technique is able to provide the chemical composition of the fat species in the tissue. Beattie *et al.* reported the use of Raman spectroscopy in the evaluation of the amount of fat and its degree of unsaturation in adipose tissues. Quantitative calibration models for adipose tissues were constructed with *R*^2^ values of 0.94–0.97 using Raman spectroscopy^7^. Raman spectroscopy has also been used for non-invasive quantitative analysis of the unsaturation index of white adipose tissues^8^. The variation of lipid content in the upper layer of the skin has been analyzed using Raman spectroscopy. The effect of hydration on the physiological properties of ceramide in the stratum corneum has also been studied with Raman spectroscopy^9^. The quantitative analysis of lipid content in the outermost layer of skin was reported by Vyumvuhore *et al.* The stratum corneum lipids such as ceramide, free fatty acids and cholesterol, have been determined using partial least square regression (PLSR) analysis with an *R*^2^ value of 0.99^10^. The HDL and LDL study in human serum was reported using near infrared spectroscopy and titanium oxide (TiO_2_) beads to immobilize the cholesterol components. PLSR models were used for the prediction of concentration of HDL and LDL with r^2^ of 0.99^11^,^12^.

The main purpose of this study was to demonstrate the ability of fiber-optic Raman spectroscopy to quantitatively analyze the molecular composition of fat in subcutaneous adipose tissues under the skin in a totally non-invasive manner. In our previous work, a fiber-optic Raman probe was used to direct measurement of the fat composition in epidermis - dermis layer (≈40 μm) but it was not possible to obtain sufficient quantitative result because the contribution of fat is much lower than that of skin (collagen, keratin, blood). In the present work, the key was an improvement in the fiber-optic Raman probe. A ball lens top hollow optical fiber Raman probe (BHRP) was modified and optimized to detect fat signals deep into subcutaneous layer, about 400 μm beneath the skin surface^13^,^14^,^15^. Moreover, the spectral analysis was improved to determine concentrations of fats with different chain lengths in the skin, eliminating the strong contributions of other materials such as proteins and blood. The Raman analysis focused on two lipids, tricaprin (TC) and trilinolein (TL), which their difference in chain lengths and degrees of unsaturation could affect the lipid accumulation/metabolism. Their accumulation rates in adipose tissues were estimated in relation to the fat treatments. The transportation of fat between the skin, SAT and VAT was evaluated in order to determine lipid accumulation and the metabolic pathways in a quantitative manner.

## Results and Discussion

### Qualitative analysis of lipid accumulation

**Figure 1:**
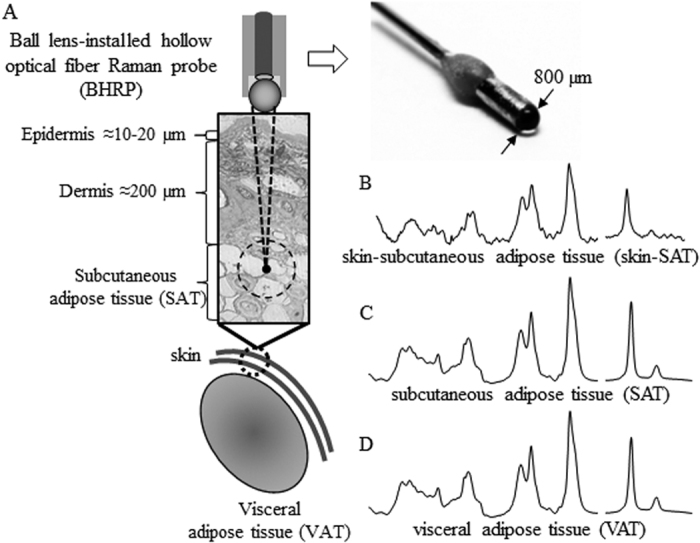
Schematic and photo of the BHRP with an 800 μm quartz ball lens (**A**). Skin spectrum (**B**), subcutaneous adipose tissue spectrum (**C**), and visceral adipose tissue spectrum (**D**).

**Figure 2:**
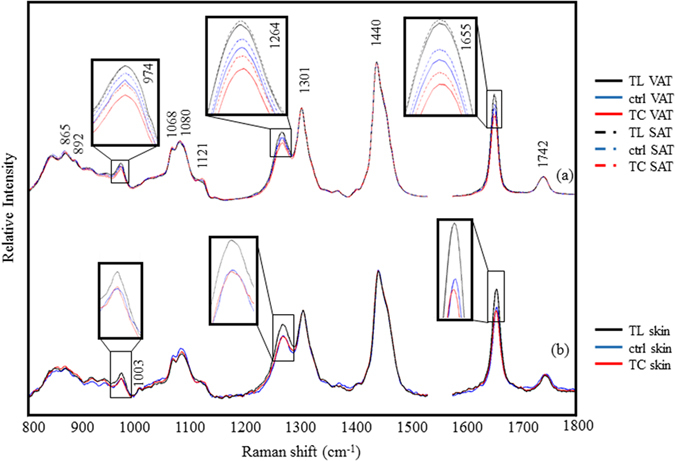
Raman spectra of (**a**) the VAT and SAT and (**b**) the skin of the animals after six weeks of dietary fat treatment. The spectra of hamsters treated with different fatty acids are shown as follows: control (blue), TC (red) and TL (black).

Figure 1 shows the experimental schematic for the non-invasive Raman measurements. The BHRP with an 800 μm fused-silica ball lens was designed to recover information from the subcutaneous layer. Its WD is estimated to be approximately 400 μm in air and 600 μm in water according to ray tracing. The effective diameter of the lens to the parallel light was about 600 μm which is enough larger than the inner diameter of the hollow fiber (320 μm). Incident light out of this diameter is reflected by total reflection. The thickness of the hamster skin was estimated to be 220–240 μm from the epidermis to the subcutaneous layer through a histological study. Therefore, the skin spectra measured with the BHRP present strong contributions of the adipose tissues beneath the skin. The Raman spectra of the dissected SAT, VAT, and skin of hamsters treated for six weeks are compared in Fig. 2. The SAT and VAT spectra show a large variation in the spectral region near 1655, 1260, and 974 cm^−1^. Contributions due to proteins and blood are not observed, indicative of a very high concentration of lipids in the adipose tissues. Since VAT is found behind the muscles in the abdominal cavity, and SAT is beneath the dermis, it is difficult to obtain information on these tissues with normal optical methods without having to perform surgery. The spectra of the skin fat are depicted in Fig. 2(b). Although bands due to collagen and the dermis layer can be observed in the low frequency region, the spectra show similarity with those of the adipose tissues. The bands at 973 and 1003 cm^−1^ are assigned to collagen and phenylalanine, respectively. The band at 1742 cm^−1^ is assigned to the C=O stretching mode of the ester groups in the glycerol heads of TAG. The weak bands in the low frequency region (1121, 1080, 1068, 892, and 865 cm^−1^) are assigned to the C–C bending modes of the lipid skeleton. The band at 974 cm^−1^ is attributed to the =C–H bending mode in unsaturated fatty chains. The dietary fat treatments resulted in major differences in the total number of double bonds, whose bands are observed at 1655, 1264, and 974 cm^−1^. The changes in the patterns of these bands reflect very well the type of dietary fat. TL is categorized as a long-chain fatty acid (LCFA) with an 18 carbon chain and two unsaturated bonds. The bands corresponding to the double bonds are relatively more intense in the TL-treated samples than those of the control and TC-treated samples. In contrast, TC is categorized as a medium chain fatty acid (MCFA) and its chain contains 10 carbons with no unsaturated bonds. The intensity of the bands assigned to the double bonds is lower in the spectra of all the tissues of the animals treated with TC. The bands of the control group spectra are found half way between TL and TC spectra. The spectra suggest that SAT typically contains more double bonds than VAT for every sample. The difference of the double bond band intensity between SAT and VAT is smaller in the samples treated with TL than in the control and TC-treated samples. The same trend was observed in all the animals (*n* = 24) tested in the present study.

The normalized skin fat spectra in Fig. 2(b) show similar features to those of the SAT and VAT spectra, due to the strong contributions from the fat. Skin fat spectra were obtained with the BHRP (WD 400 μm) from deeply beneath the dermis, in the subcutaneous layer, in a totally non-invasive manner. The major differences between the SAT and VAT spectra are observed in the bands at 855, 874, and 1003 cm^−1^, assigned to collagen and phenylalanine. Although the spectral features in these bands show variation compared to those of the skin fat spectra, this may not be due to the dietary fat but to differences in the local thickness of the epidermis and dermis layers. In contrast, the variation in intensity of the bands at 1655, 1264, and 974 cm^−1^, ascribed to the double bonds, seems to be related to the diet treatments, especially in the case of TL. The variation between the control and TC samples is relatively small, since the amount of TC accumulation is much lower than that of TL. Moreover, the spectra of fat obscured by protein, blood and so on in the skin tissue made it even more difficult to analyze. To confirm that the differences in the spectra are due to the dietary fat treatments, multivariate analysis was applied to extract the treatment-related spectral changes from other random variables and the noise of the spectra. The spectral intensity of the double bond bands is sensitive to lipid oxidation. The oxidation process, which is particularly important in lipids with multiple double bonds, initiates through the delocalization of the double bonds (C=C), which move along the fatty chain and result in the construction of conjugated systems. This is followed by a peroxidation reaction with oxygen. According to our previous study, the conjugated dienes in linoleic acid show a much stronger band due to the C=C stretching mode, which is slightly shifted to 1655 cm^−1^, whereas the intensity of the band at 1264 cm^−1^ remains unchanged with respect to that of non-conjugated linoleic acid. The similar enhancement of the band at 1655 cm^−1^ have been observed in the air oxidation of linoleic acid, where the intensity of the band at 1655 cm^−1^ increased and no change was observed in the band at 1264 cm^−1^ in the earlier stage of oxidation process^15^,^16^. Since the intensity ratio between the bands at 1655 and 1264 cm^−1^ (*I*_1655/1264_) becomes remarkably large when the fat includes conjugated double bonds, the extent of lipid oxidation can be estimated through the value of *I*_1655/1264_.

### PCA of SAT and VAT samples

**Figure 3:**
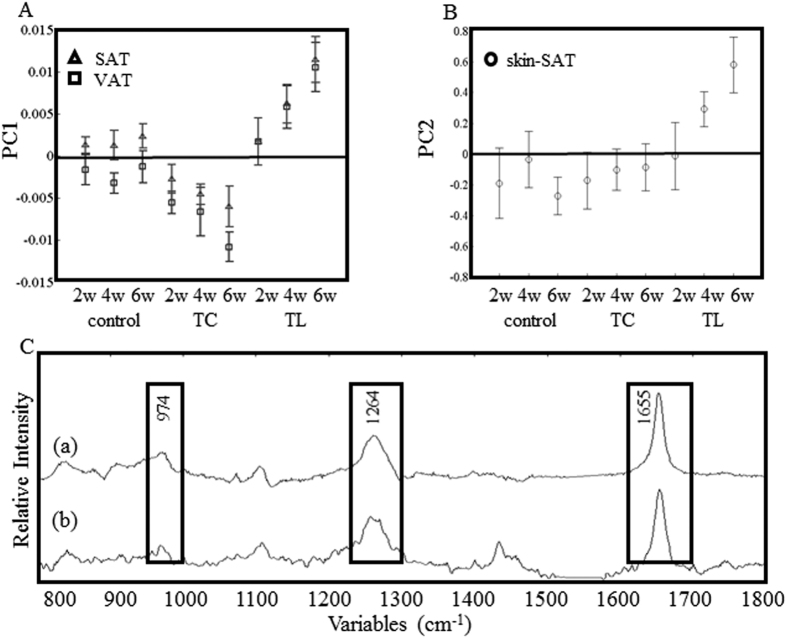
PCA analysis results for the VAT, SAT, and the skin spectra of the animals subjected to dietary fat treatment. Score plots for PC1 (**A**) of VAT (□) and SAT (Δ) datasets and those for PC2 (**B**) of the skin dataset.

PCA was performed to analyze the characteristic variation of the spectra as a result of the fat composition. SAT and VAT (*n* = 360) spectra were collected from 24 animals in total, which included the TC- and TL-treated and control groups. PC1 (57% explained variance) displayed the largest variation regarding the dietary fat. The PC1 scores are plotted for the samples after 2, 4, and 6 weeks of diet treatment in Fig. 3A. The loading plot of PC1 (Fig. 3C(a)) depicts strong bands at 1655, 1264, and 974 cm^−1^, which are assigned to the C=C stretching, =C–H bending, and out-of-plane *cis* mode =C–H bending, respectively. This suggests that PC1 strongly reflects the total number of double bonds in a sample. According to the score plot, the concentration of double bonds in the TC-fed sample group decreases, although it increases in the TL-fed group. This indicates that the double bond rich TL and the saturated TC absorbed through the digestive organs are transferred into adipose tissues in the body with minimum modification of their structures, i.e. the fatty chains are not decomposed in the process. SAT values always display a slightly higher concentration of double bonds than VAT. It has also been reported in previous papers that SAT presents less saturated fatty acids than VAT^17^,^18^.

The PCA results for the skin fat spectra measured through the skin are different from those of dissected tissues. The largest variations in PC1 arise from protein rich tissues, while PC2 reflects the variation in fat composition. The loading plot of PC2 (10% explained variance) of skin fat in Fig. 3C(b) shows a similar spectral feature to that in PC1 of VAT and SAT datasets. The scores of PC2 are plotted in Fig. 3B, suggesting changes in the double bonds of the skin fat samples under dietary control. The reduction of double bonds is, however, indistinguishable in the TC-treated groups. This is possibly due to interferences from the skin, which make the Raman signals too weak to analyze. It is therefore not possible to detect the accumulation of TC in the samples through PCA. The changes in the C–C backbone of fatty acids must be taken into consideration in order to detect specific fats like TC^15^.

### Quantitative analysis of specific fats with PLSR analysis

**Figure 4:**
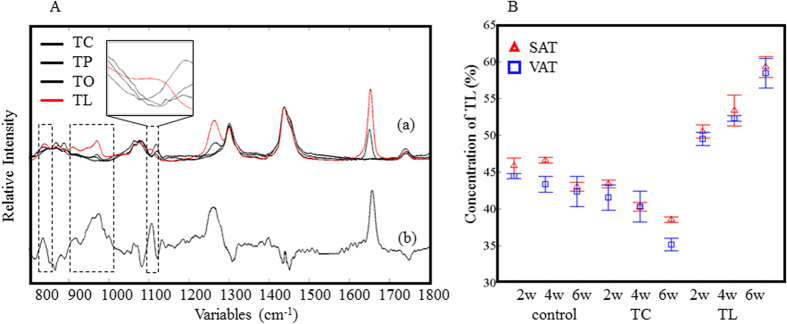
Raman spectra of pure fatty acids (a) and regression coefficients (b) from the PLSR calibration model for TL of SAT and VAT (**A**). The concentration of TL in the VAT (□) and SAT (Δ) of the animals was estimated from the Raman spectra (**B**).

**Figure 5:**
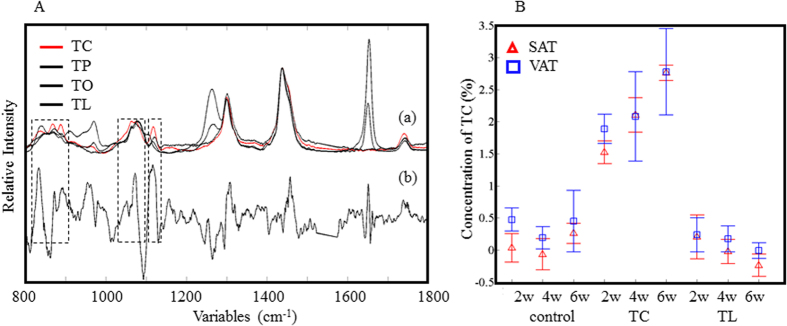
Raman spectra of pure fatty acids (a) and regression coefficients (b) from the PLSR calibration model for TC of SAT and VAT (**A**). The concentration of TC in the VAT (□) and SAT (Δ) of the animals was estimated from the Raman spectra (**B**).

In order to observe changes in specific fats, the Raman spectra of SAT and VAT were subjected to a PLSR model. The relative concentration of TL and TC over the total fats was determined by GC in each sample and used as dependent variables in the PLSR analysis. The GC measurements were carried out in triplicate for 24 samples in each experiment (SAT and VAT, 12 animals each). About 15 Raman spectra were obtained from each sample. The datasets were separated into two groups: a calibration set (*n* = 180) and a validation set (*n* = 180), and the prediction model built with the former was verified with the latter. The PLSR model for TL was built with four factors and its correlation coefficient (*R*^2^ value) was 0.91. The *R*^2^ value suggests that the model is highly reliable. On the other hand, the model for TC was built with six factors and its *R*^2^ value was 0.83. In the PCA results, the bands due to the double bonds included information on TC and TL as well as other fats, such as palmitic acid, oleic acid and linolenic acid, as confirmed by GC. The PLSR analysis successfully excluded the interference of those unwanted fats. The regression coefficients of the TL calibration model were compared to the spectra of TL-, TC- and two other common fats, tripalmitin (TP)- and triolein (TO)-treated samples, as shown in Fig. 4A. The bands at 1655 and 1264 cm^−1^ (related to the double bonds) were also observed in the PC1 loading plot of the PCA (Fig. 3C(a)). In contrast, the broad bands near 815–850, 902–1010, and 1092–1155 cm^−1^ (indicated with boxes) only appear in the PLSR analysis. These bands are also observed only in the spectra of the TL-treated samples. The concentration of TL in the test samples estimated by the PLSR calibration model is shown in Fig. 4B. Consistent with the PCA results, SAT yields a higher concentration of TL (the unsaturated fat) than VAT. It should be noted that the TL concentration varied from 35% to 60%. This indicates that the fat composition has large flexibility and depends highly on the dietary fat composition. The regression coefficients of the TC calibration model are shown in Fig. 5A. The bands in the low frequency region appear stronger than those assigned to the C–H and C=C vibrational modes. This suggests that the bands in the low frequency region are important in order to discriminate fats with different chain lengths. The plot of the regression coefficients for the TC analysis is noisier than that for the TL analysis. This is because the concentration of TC in SAT and VAT is naturally small, less than 3%. The concentration of TC in SAT and VAT, as estimated by the PLSR calibration model for TC, is depicted in Fig. 5B. The concentration of TC is almost zero in the control samples since mid-chain fats, including TC, are not synthesized in the body. However, TC was found in the adipose tissue of the TC-treated group. This indicates that there is a path for TC or the decanoic acid chain to be directly absorbed and accumulated in adipose tissues. The TC uptake is, however, less susceptible in comparison with the TL uptake. The present PLSR analysis results demonstrate the great potential of Raman spectroscopy for the quantitative analysis of specific fats, even at quite low concentrations (0.6–2.7%).

The body weight of the hamsters was typically 110 g at the beginning of the experiment and increased up to 160 g after six weeks of fat treatment. No particular trend in the weight variation of the diet groups was observed in comparison with the control group. The experiment assumes that the fat-treated animals reduced their normal food intake by themselves, resulting in no excess weight gains in the fat diet groups. The TL concentration in the TC fed group was slightly reduced or remained unchanged during the experiment. According to the GC results, the palmitic concentration increased in the TC-treated group and decreased or remained unchanged in the TL-treated group. Palmitic fat is the primary fatty acid produced from fatty acid synthase (FAS)^19^. This suggests that TC was mostly metabolized and used as material for biosynthesis. In contrast, TL was directly absorbed and accumulated in the body.

### Non-invasive quantitative analysis of the fat composition

**Figure 6:**
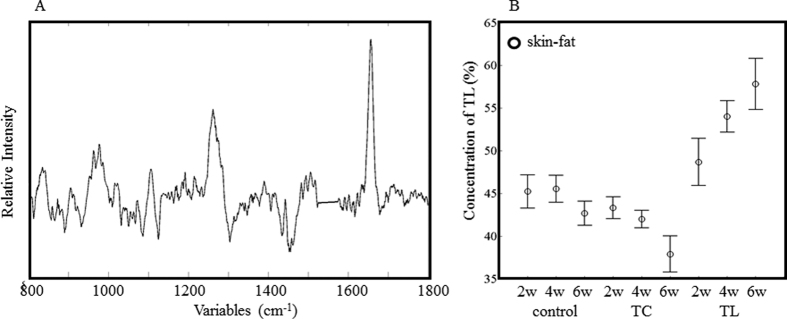
Regression coefficients of the PLSR calibration model for TL (**A**) in skin. The concentration of TL in the skin of the fat-treated animals was estimated from the Raman spectra (**B**).

**Figure 7:**
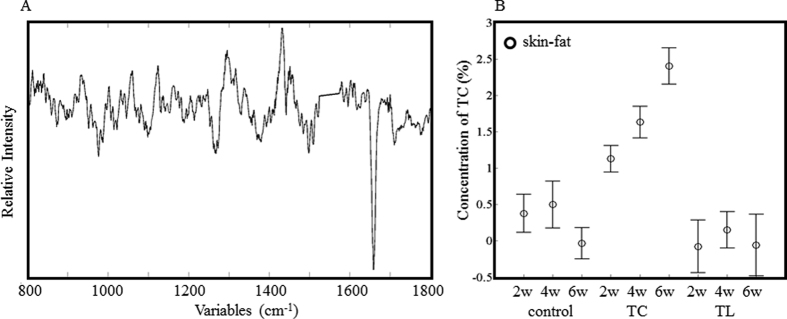
Regression coefficients of the PLSR calibration model for TC (**A**) in skin. The concentration of TC in the skin of the fat-treated animals was estimated from the Raman spectra (**B**).

The PLSR results for the skin fat spectra were successfully used to build up a calibration model for TL and TC accumulation, of which the *R*^2^ values were 0.87 and 0.77, respectively. The latent variables were five. Since it was not possible to analyze the skin fat by GC, the SAT GC data were applied for the dependent variables in order to build the PLSR models. The reliability of the model was reduced because the intensity of the skin spectra was generally weaker than that of adipose tissues, as proteins and other components of the skin interfered in the analysis. The regression coefficients of the TL calibration model are shown in Fig. 6A. Its features are similar to those of the TL calibration model for SAT and VAT in Fig. 4A, although it has a higher noise level. This implies that the model extracts successfully the properties of the TL spectra. The estimated concentrations of TL are plotted in Fig. 6B. Although the deviation of the data is larger than that for SAT, the data show a similar trend. The regression coefficients for skin fat (Fig. 7A) show, to some extent, a similar pattern to those of SAT, especially in the low wavenumber region (800–1200 cm^−1^). The bands in the low frequency region provide a characteristic profile for the specific fats. The estimated TC concentrations plotted in Fig. 7B also show a pattern similar to that of SAT. It is noteworthy that PLSR analysis was able to extract information of TC even in the skin spectra, whereas PCA was not.

The present results suggest that the working distance of BHRP, about 400 μm, is long enough to reach the subcutaneous layer. There is a boundary between the dermis and the subcutaneous layer at 245 μm, beneath the surface of the hamster skin. The diameter of the sampling volume was estimated to be more than 100 μm, which seems to include both regions and consists of not only fat, but also proteins, sugars, and blood. The skin spectra measured with the BHRP, however, shows a high contribution of fat, suggesting that Raman spectroscopy is highly sensitive to fat species. It is also suggested that the present method is able to identify and to quantitatively determine specific fats at concentrations as low as 2.7%.

In the present study, the predicted concentrations of TL and TC were similar in SAT and skin, a natural consequence since the model was constructed with the same dependent variables from the GC data. However, it must be noted that the predicted values represent validation results of unknown samples. Hence, these results demonstrate that the BHRP system is able to obtain information of subcutaneous fat in a completely non-invasive manner, and that Raman spectroscopy is able to select information of specific fats from skin spectra.

### Accumulation rates of TL and TC

The accumulation rates of TL and TC in SAT, VAT, and the subcutaneous tissue were calculated from the PLSR predicted values and are shown in Table 1. The rates for TL and TC reflect very well the characteristic metabolism for long and medium chain fatty acids. There are no significant differences between SAT and VAT in terms of the accumulation rate, suggesting that the fat metabolism system is comprehensive for both adipose tissues. In contrast, the accumulation ratio of TL is approximately 8.01 times higher than that of TC. This shows that the metabolic pathways are different for TL and TC. This is probably ascribed to the difference in their chain length; TC has 10 carbons (a medium-chain fat) while TL has 18 carbons (a long-chain fat). Medium-chain fats are generally sent through the blood stream directly to the liver and broken down into acetyl-CoA, whereas long-chain fats are incorporated into chylomicrons, which are then absorbed by peripheral organs and sent to adipose tissues^4^,^20^,^21^. Long-chain fats are also converted into energy, although this requires carnitine palmitoyltransferase (CPT), which regulates the rate of consumption of long-chain fats^22^. Hence, the present results demonstrate that it is possible to estimate the relationship between the state of a metabolic system and the dietary fats from the subcutaneous skin fat composition measured by fiber-optic Raman analysis.

## Conclusions

This study demonstrates the feasibility of using fiber-optic Raman spectroscopy in the quantitative analysis of specific fats in the body in a totally non-invasive manner. The subcutaneous adipose tissue underneath the skin was successfully determined by a BHRP with an 800 μm fused-silica ball lens, specially designed for subsurface measurements. Interestingly, we found that the unsaturation degree of SAT was always higher than that of VAT, regardless of the fat diet. PLSR calibration models for TL and TC were successfully built for the quantitative analysis of the Raman spectra along with the GC data. The dietary fat treatment animal study indicated the quick decomposition of TC and quick accumulation of TL, suggesting differences in the metabolic pathways for different chain-length fats. The present results obtained from Raman analysis are in good agreement with previous fat studies, demonstrating the great potential of fiber-optic Raman spectroscopy for fat metabolism studies, as well as for dietary fat control for the maintenance of good health.

## Materials and Methods

### Preparation of the animal models

Golden Syrian hamsters were purchased at six weeks old (*n* = 24) (SLC, Shizuoka, Japan). They were assigned to 3 treatment groups: one fed with distilled water (control), a second one fed with TL (TCI, Tokyo, Japan), and a third one with TC (TCI, Tokyo, Japan). The supplements were prepared daily to avoid degradation and denaturation, and directly introduced into the stomach with an oral gavage needle once a day. The amount of supplement given was 0.5% of each hamster’s body weight. The supplements were administered every day for six weeks. Normal food (Picolab Rodent Diet 5053 (LabDiet^®^, St. Louise, MO, USA) and water was unrestricted to the animals at all times. The Raman measurements of the skin were conducted under anesthesia. Then, the animals were culled and autopsied after the six-week experiment and samples of VAT and SAT were obtained for one further measurement. The dissected tissues were kept at −80 °C to prevent lipid oxidation. The experiments were performed in accordance with relevant of the approved guidelines and regulations of the Ethics Committee of Kwansei Gakuin University.

### Raman measurements

The homemade BHRP consisted of an 800 μm fused-silica ball lens (Edmund Optics, USA) and a hollow optical fiber of 420 μm of outer diameter (Doko Engineering LLC, Japan). The working distance (WD) was estimated using ray tracing method. The WD was estimated to be 400 and 600 μm in air and water for the paraxial light. The WD is slightly shorter for the incident light far from the lens center because of spherical aberration. The effective diameter was about 600 μm which was restricted by total reflection angle in the lens. A diode laser emitting at 785 nm (Toptica Photonics, Germany) was used as the excitation source for the Raman measurements. A single polychromatic Raman spectrometer (F4.2, focal length 320 mm, 750 nm blazed, 600 l mm^−1^ grating; Photon Design Co. Ltd., Japan) and a charge-coupled device detector (DU420-BRDD; Andor Technology Co. Ltd., Northern Ireland) were used to record the Raman spectra. The BHRP was coupled to the spectrometer through a coupling stage which had a long-pass filter (LF; Semrock, USA), a notch filter (NF; Kaiser Optical System, USA), and two lenses. The Raman scattered light transferred by the hollow optical fiber was finally focused onto the slit (100 μm width) of the spectrometer. The samples were measured after two exposures with a 60 mW excitation light for 30 s. After background subtraction, the baseline undulation was corrected with a 5^th^ polynomial line fit. The spectra were normalized using the band at 1440 cm^−1^. Spectral pretreatment was carried out using MATLAB (Mathworks Inc., MATLAB Version 7.1 R2010a). Principal component analysis (PCA) with leave-one-out cross validation and partial least square regression (PLSR) analysis were carried out with Unscrambler 10.1 (CAMO Software AS., Oslo, Norway), which was used to construct the calibration model.

### Lipid extraction for gas chromatography (GC)

The lipids in VAT and SAT were extracted using the Bligh and Dyer method^23^. The collected adipose tissue (300 mg) was mixed with chloroform/methanol/dH_2_O (1:2:1 v/v) and sonicated six times for 10 s (each time). The chloroform–lipid layer was then dried under N_2_. The extracted lipids were methylated with a methylation kit (Nacalai Tesque, Kyoto, Japan). A methyl heptadecanoate solution (20 μl of 0.02 g/300 μl toluene) was added as an internal standard. Gas chromatography coupled with a flame ionization detector was used to measure the fatty acid methyl esters (GC-2010, Shimadzu Co., Ltd., Kyoto, Japan). The injector port and detector were set to 250 °C. The oven temperature was programmed to increase from 90 °C to 240 °C at 5 °C/min. The peak area of each lipid species was normalized with respect to the internal standard. The amount of fatty acids was calculated from a gas chromatography calibration curve of standard fatty acids (decanoic acid, palmitic acid, oleic acid, and linoleic acid), which were purchased from Sigma-Aldrich (St. Louis, MO, USA) and used without further purification.

## Additional Information

**How to cite this article**: Meksiarun, P. *et al.* Non-invasive Quantitative Analysis of Specific Fat Accumulation in Subcutaneous Adipose Tissues using Raman Spectroscopy. *Sci. Rep.* **6**, 37068; doi: 10.1038/srep37068 (2016).

**Publisher’s note:** Springer Nature remains neutral with regard to jurisdictional claims in published maps and institutional affiliations.

## Figures and Tables

**Table 1:** Accumulation rates of TL and TC in VAT, SAT and skin per two weeks.

Average accumulation rate (%) per 2 weeks		
Trilinolein in TL-treated group	visceral adipose tissue (VAT)	4.45 ± 1.6
	subcutaneous adipose tissue (SAT)	4.37 ± 2.4
	skin-subcutaneous (skin-SAT)	4.57 ± 3.41
Tricaprin in TC-treated group	visceral adipose tissue (VAT)	0.58 ± 0.4
	subcutaneous adipose tissue (SAT)	0.52 ± 0.38
	skin-subcutaneous (skin-SAT)	0.63 ± 0.3
